# Minimally Invasive Versus Open Laminectomy/Discectomy, Transforaminal Lumbar, and Posterior Lumbar Interbody Fusions: A Systematic Review

**DOI:** 10.7759/cureus.1488

**Published:** 2017-07-18

**Authors:** Allicia O Imada, Tridu R Huynh, Doniel Drazin

**Affiliations:** 1 College of Medicine, University of Vermont; 2 Department of Neurosurgery, Cedars-Sinai Medical Center

**Keywords:** systematic review, minimally invasive spine surgery, neurosurgery

## Abstract

Minimally invasive spine surgeries (MISS) are becoming increasingly favored as alternatives to open spine procedures because of the reduced blood loss, postoperative pain, and recovery time. Studies have shown mixed results regarding the efficacy and safety of minimally invasive procedures compared to the traditional, open counterparts. The objectives of this systematic analysis are to compare clinical outcomes between the three MISS and open procedures: (1) laminectomy/discectomy, (2) transforaminal lumbar interbody fusion (TLIF), and (3) posterior lumbar interbody fusion (PLIF). The Cochrane and PubMed databases were queried according to the preferred reporting items for systematic review and meta-analyses (PRISMA) statement. The primary outcome measures included the visual analog scale (VAS), the Oswestry disability index (ODI), and blood loss. A total of 32 studies were included in the analysis. Of the three procedures investigated, only MISS TLIF showed significantly improved VAS for leg pain (p = 0.02), ODI (p = 0.05), and reduced blood loss (p = 0.005). MISS-laminectomy/discectomy, TLIF, and PLIF appear to be similar in terms of postoperative pain and perioperative blood loss. MISS TLIF is perhaps more effective in specific outcome measures and results in less intraoperative blood loss than open TLIF.

## Introduction and background

In recent years, minimally invasive spine surgery (MISS) has become an increasingly attractive alternative to open spine surgery because of a combination of technological advances and a continued desire to reduce tissue injury, complications, and recovery time through the use of minimal incisions and specialized instruments [1-2]. First introduced in 1997 by Foley and Smith for the microscopic decompression of spinal stenosis, MISS is now being applied to a broad spectrum of pathologies, including, but not limited to, adult spinal deformities, trauma, and malignancies [3-6]. In the surgical treatment of lumbar stenosis and degenerative lumbar spondylolisthesis, MISS procedures, including unilateral laminotomy, bilateral laminectomy for bilateral decompression, and transforaminal lumbar interbody fusion (TLIF) have become popular procedures [4,7,8]. Posterior lumbar interbody fusion (PLIF) is another procedure that can be performed using minimally invasive techniques [9].

Despite the widespread and accepted use of MISS, many surgeons still question their safety compared to their traditional, open counterparts. Three criteria have been put forward to evaluate this point: (1) equal or superior treatment of symptoms; (2) reduction in perioperative tissue trauma, physiologic stress, and disturbance of biomechanics; and (3) reduction in complications, infections, and need for subsequent surgeries [3]. With regard to the first criteria, a review by Skovrlj et al. compared the minimally invasive versus the open procedure for laminectomy, TLIF, and direct lateral interbody fusion (DLIF). The authors reported MISS to be as effective as the analogous open procedures [10]. With respect to the second criteria, MISS procedures have also been shown to decrease injury to the multifidus muscle [2], decrease physiologic stress [11], as well as maintain the biomechanical properties of the spine [12].

Controversy remains, however, regarding the third criteria: perioperative outcomes. While studies addressing this concern are available for a number of these procedures, many are inherently limited in their design as prospective/retrospective cohort studies or national database analyses [13-16]. Recently, however, a number of randomized and nonrandomized clinical trials comparing minimally invasive to open procedures have been published [14-23]. In an effort to more accurately characterize the effectiveness of MISS versus open analogs, we conducted a systematic review looking at the perioperative and postoperative outcomes for three spine procedures: (1) laminectomy/discectomy, (2) TLIF, and (3) PLIF. 

## Review

Study Inclusion

**Figure 1:**
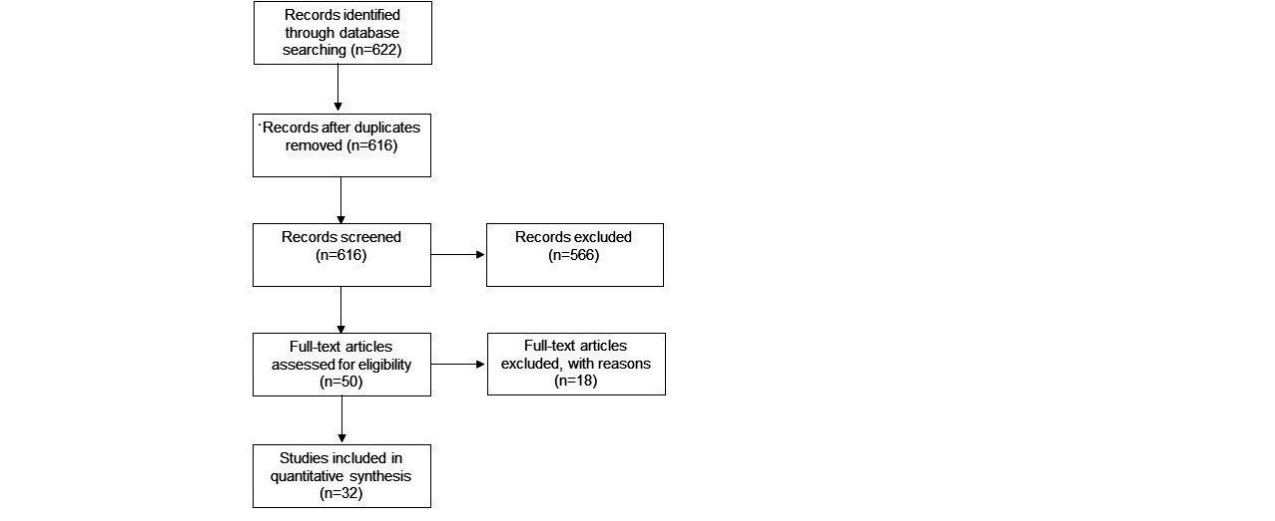
Flowchart According to the PRISMA Statement The preferred reporting items for systematic reviews and meta-analyses (PRISMA) checklist was followed for study selection and the 2009 flow diagram is shown.

The preferred reporting items for systematic reviews and meta-analyses (PRISMA) guidelines were adhered to throughout this study. Our workflow is summarized in Figure 1. This systematic review is registered under the PROSPERO International prospective register of the National Institute for Health Research (CRD42017060375). Institutional review board approval was not required for this study. Electronic searches of the Cochrane Library and PubMed databases were performed by two independent authors (AI and TH) through November of 2016. Strategic search term combinations were utilized and included "minimally invasive" and "spine" and "surgery" and "outcomes." English, full-text clinical studies/trials involving human adults ages 19 and over were included. The title and abstract views were screened for relevance to the topic and duplicate articles were removed. A total of 32 quantitative studies were included in the analysis. Procedures were categorized based on their description in individual studies as open or MISS laminectomy/discectomy, TLIF, or PLIF.

Outcome Measures

The postoperative values were recorded at the final follow-up for each study, and these included the visual analog scale (VAS) for leg pain and the Oswestry disability index (ODI). VAS scores were scaled across studies to be 0-10 centimeters, to allow for comparison. Estimated intraoperative blood loss was also recorded. We focused on these three outcome measures because they were the most prevalent across the studies analyzed.

Statistical Analysis

Descriptive statistics were ascertained for the included studies. Means and standard deviations were calculated for all outcomes of interest. Paired, parametric t-tests and single factor analyses of variance (ANOVAs) were used to evaluate for significant differences between procedural groups using IBM Statistical Package for the Social Sciences (SPSS), version 23.

Laminectomy/Discectomy

A total of 18 studies were identified involving open and MISS laminectomy/discectomy: 12 analyzed MISS laminectomy/discectomy, 1 analyzed open laminectomy/discectomy, and 5 compared open versus MISS laminectomy/discectomy (Table 1) [15-18,21,24-36]. The mean follow-up time was 20.47; range: 12-40.2 months with an average of 119.44; and range: 8-721 patients. There were no significant differences in terms of VAS for leg pain (mean = 4.56 ± 1.04 vs. 4.58 ± 0.96, p = 0.98); no significant difference in ODI (mean = 31.84 ± 11.30 vs. 17.40 ± 0.57, p = 0.10); and no significant difference in intraoperative blood loss (mean = 70 ± 51 vs. 139 ± 71, p = 0.10; Table 2).

**Table 1:** Characteristics of Included Studies RCT: randomized controlled trial; RC: retrospective cohort; PC: prospective cohort; PELD: percutaneous endoscopic lumbar discectomy; PDD: percutaneous disc decompression; PLDD: percutaneous laser disc decompression; physio: physical therapy; X-STOP: interspinous process decompression system; PEDTA: posterolateral transforaminal selective endoscopic discectomy and thermal annuloplasty; PCS: percutaneous cervical discectomy; PCN: percutaneous cervical disc nucleoplasty; PCDN: percutaneous cervical discectomy and nucleoplasty; LDH: lumbar disc herniation; LSS: lumbar spinal stenosis; discogenic lower back pain; cervical disc herniation; sacroiliac joint dysfunction; DS: degenerative spondylolisthesis; IS: isthmic spondylolisthesis; DDD: degenerative disc disease; TJF: thoracolumbar junction fractures; PS: post laminectomy syndrome; CLBP: chronic lower back pain

Authors & Year	Study Type	Included Cases	Diagnosis	Follow-Up (months)	Technique Used
Laminectomy/Discectomy					
Ying et al., 2006 [24]	RCT	45	LDH	12	PELD
Nikoobakht et al., 2015 [25]	RCT	177	LDH	12	PDD vs. physio
Nerland et al., 2015 [15]	RC	721	LSS	12	Microdecompression vs. open laminectomy
Brouwer et al., 2015 [17]	RCT	115	LDH	12	PLDD vs. open discectomy
Lonne et al., 2015 [26]	RCT	96	LSS	24	Microdecompression vs. X-STOP
Cheng et al., 2014 [27]	PC	113	LDH	36	PEDTA
Mobbs et al., 2014 [18]	RCT	54	LSS	40.2	MI vs open laminectomy
Yang et al., 2014 [28]	RC	171	CDH	40.2	PCS vs. PCDN vs. both
Majeed et al., 2013 [16]	RC	66	LDH	24	Microdecompression vs. open discectomy
Wong et al., 2012 [29]	CS	17	LSS	12	Mild interlaminar decompression
Gerszten et al., 2010 [30]	RCT	90	LDH	12	PDD vs. epidural corticosteroids
Yagi et al., 2009 [21]	RCT	41	LSS	18	Microdecompression vs. open laminectomy
Pao et al., 2009 [31]	PC	53	LSS	16	Microendoscopic laminotomy
Matsumoto et al., 2007 [32]	PC	36	LDH	21	Microdiscectomy
Dewing et al., 2008 [33]	PC	197	LDH	26	Microdiscectomy
Cho et al., 2007 [34]	RCT	70	LSS	15	Open laminectomy vs. marmot operation
Sasaki et al., 2006 [35]	PC	8	LSS	24	Laminotomy
Kim et al., 2007 [36]	RCT	80	LSS	12	Laminotomy
		Mean = 119.44; range [8 – 721]		Mean = 20.47; range [12 – 40.2]	
Transforaminal Lumbar Interbody Fusion					
Gu et al., 2015 [12]	PC	74	SIJD	32	MI-TLIF
Shen et al., 2014 [37]	RCT	65	DDD	27	MI-TLIF
Nandyala et al., 2014 [38]	RCT	52	LSS, DS	12	MI-TLIF
Perez-Cruet et al., 2014 [39]	PC	304	IS, DS, LSS, LDH	47	MI-TLIF
Choi et al., 2013 [40]	RCT	53	DDD	28	MI-TLIF
Rodriguez-Vela et al., 2013 [41]	PC	41	DDD	45	open-TLIF
Tsahtsarlis et al., 2012 [42]	PC	34	DDD	28	MI-TLIF
Wang et al., 2014 [20]	NRCT	81	LSS, DS, IS, PS	12	MI vs. open-TLIF
Sembrano et al., 2016 [43]	RCT	55	DS, LSS	24	MI-TLIF
Gandhoke et al., 2016 [23]	PC	74	DS	24	MI vs. open-TLIF
Wang et al., 2011 [19]	RCT	79	DDD	24	MI vs. open-TLIF
		Mean = 82.91; range [34 – 304]		Mean = 27.54; range [12 – 47]	
Posterior Lumbar Interbody Fusion					
Li et al., 2015 [14]	PC	30	TJF	24	MI vs. open-PLIF
Song et al., 2015 [44]	PC	54	IS	27	Open PLIF
Kasis et al., 2009 [22]	PC	323	CLBP, DS	24	MI vs. open-PLIF
		Mean = 135.67; range [30 – 323]		Mean = 25; range [24 – 27]	

**Table 2:** Summary of Analysis Comparing Outcome Measures in Open Versus Minimally Invasive Procedures * indicates p-value < 0.05

Outcome Measures	Minimally Invasive – Laminectomy/Discectomy	Open –Laminectomy/Discectomy	p -value	Minimally Invasive –Transforaminal Lumbar Interbody Fusion	Open – Transforaminal Lumbar Interbody Fusion	p -value	Minimally Invasive – Posterior Lumbar Interbody Fusion	Open – Posterior Lumbar Interbody Fusion
VAS for leg pain			0.98			0.02*		
Mean	4.56	4.58		5.36	3.75		5.1	4
SD	1.04	0.96		0.85	0.74		0	0.3
ODI			0.10			0.05*		
Mean	31.84	17.40		24.21	17.20		28.6	36.57
SD	11.13	0.57		5.52	5.94		0	12.76
Blood loss			0.13			0.005*		
Mean	70	139		158	452		323	595
SD	51	71		77	273		0	93

While we found no statistical differences in the three outcome measures of interest between open and MI laminectomy/discectomy, the raw difference seems to be considerable with regards to ODI, yet fails to reach statistical significance (ODI mean = 31.84 ± 11.30 vs. 17.40 ± 0.57, p = 0.10). This is most likely because of a lack of statistical power, as only two studies reported ODI. A recent review by Phan et al. in 2016 showed significantly improved VAS scores and reduced blood loss [45]. Contrastingly, Skovrlj et al. in 2015 reported no significant difference in blood loss between MISS and open laminectomy [10]. Nerland et al. in 2015 and Mobb et al. in 2014 showed equivalence between MISS and open laminectomy ODI scores [15,18]. Further, higher-powered, randomized controlled trials are necessary to determine whether or not MISS laminectomy/discectomy is superior to its open counterpart.

Transforaminal Lumbar Interbody Fusion

A total of 11 studies involving TLIF were identified: 1 analyzed open TLIF, 7 analyzed MISS TLIF, and 2 compared open vs. MISS TLIF [12,19-20,23,37-43]. The mean follow-up time was 27.54; range: 12-47 months on an average with 82.9; and range: 34-304 patients. MISS TLIF had significantly improved VAS scores for leg pain compared to open TLIF (mean = 5.36 ± 0.85 vs. 3.75 ± 0.74, p = 0.02; Table 2). The improvement in ODI was significantly greater in MISS TLIF (mean = 24.21 ± 5.52 vs. 17.20 ± 5.94, p = 0.05). MISS TLIF showed significantly reduced average blood loss compared to open TLIF (mean = 157 ± 77 mL vs. 452 ± 273 mL, p = 0.005).

Our significant findings are consistent with other systematic reviews comparing open and MI TLIF procedures. Goldstein et al. reported significantly reduced blood loss and ODI in the MI TLIF and PLIF procedures [46]. Similarly, Skovrlj et al. also reported significantly reduced blood loss in MISS procedures [10]. TLIF can be approached in several different ways, which include a midline incision with a Taylor retractor (Sklar Instruments, West Chester, PA), the Wiltse approach with and without a tubular retractor, and endoscopic TLIF. Neither of the above reviews nor we in this present study control for these differences, which may account for differences in our findings. Further targeted studies should compare these different approaches.

Posterior Lumbar Interbody Fusion

Three studies were identified analyzing PLIF performed open (one) or comparing MISS versus open techniques (two) [14,22,44]. The mean follow-up time was 25; range: 24-27 months with 135.67; and range: 30-323 patients on an average. Of these three studies, only one of them reported one or more of our three outcome measures of interest, and, consequently, statistical analysis and comparison could not be performed for this group. However, individual studies showed significant improvements in ODI scores and VAS for leg pain, as well as estimated reduced blood loss [14]. Sidhu et al. reported decreased blood loss, shorter hospital stays, and longer operative times in the MI PLIF groups [47]. Li et al. also reported significantly reduced blood loss in the MISS PLIF group. Further randomized trials are needed to determine whether or not MISS PLIF is superior to its open counterpart.

Overall Benefits of Minimally Invasive Spine Surgery

Of the procedures evaluated, only MISS TLIF demonstrated advantages in terms of VAS for leg pain, ODI, and blood loss. Several factors have been postulated behind the benefits of MISS, including smaller portals and reduced muscle stripping, which have been shown to reduce blood loss [19-20]. One study specifically showed significantly improved T2 relaxation time of the multifidus muscle, improved average discharge amplitude, and improved frequency of the sacrospinalis muscle in the MI group [19]. Mobbs et al. showed that patients having undergone MISS consumed significantly less mean total morphine equivalents and fewer patients required opioids after MISS [18]. It is unclear, however, what factors might distinguish TLIF from discectomy and PLIF, whether it be related to procedure or study design. No significant differences in study size (p = 0.73) between all three groups were appreciated.

Limitations

The limitations of this study are inherent to systematic reviews, and we remain cognizant of them. Selection bias was a key obstacle given the range of preoperative outcome measures reported and the baseline differences in the demographics of included studies. Since we were specifically interested in comparing the effectiveness of the open and minimally invasive versions of the three surgeries of interest, we based this analysis on procedure rather than on indication. As a result, our results may have been confounded through heterogeneity among the study populations, specifically in terms of diagnosis and indication for surgery. Specific approach techniques for each procedure were not accounted for due to variation and lack of description in specific studies. Furthermore, different surgeons at different institutions performing the surgeries may have added variability to clinical outcomes. It is unclear whether VAS is comparable from study to study because it is a subjective measure. Oswestry scoring is more standardized and, consequently, presumably more robust. Reported blood loss is highly dependent on surgeons and anesthesia practitioners and, as a result, should be interpreted carefully. Finally, this study only looked at end-point outcome measures, which might have led us to miss any potential early improvement. A plethora of validated quality of life and back pain scores to evaluate recovery exists, but questions remain as to which measures are appropriate when comparing MISS to open spine procedures.

## Conclusions

This systematic review suggests that out of laminectomy/discectomy, PLIF, and TLIF, only MI TLIF may be superior to its open analog in terms of VAS score, ODI, and intraoperative blood loss. While individual studies have demonstrated advantages in favor of MISS over traditional techniques, more highly powered, randomized clinical trials are needed to establish MISS techniques as standardized treatment strategies.
